# Percutaneous cartilage injection: A prospective animal study on a rabbit model

**DOI:** 10.1186/1916-0216-42-7

**Published:** 2013-01-31

**Authors:** Olivier X Beaudoin, Andrew Mitchell, Akram Rahal

**Affiliations:** 1 Division of Otolaryngology-Head and Neck Surgery, Maisonneuve-Rosemont Hospital, University of Montreal, Montreal, Canada; 2 Department of Pathology, Maisonneuve-Rosemont Hospital, University of Montreal, Montreal, Canada

**Keywords:** Cartilage injection, Nasal dorsum, Rabbit

## Abstract

**Background:**

Cartilage grafting is a useful technique in nasal reconstruction. Implantation of a whole graft is usually done through an incision. Crushed cartilage can also be used. Injection of cartilage could be an alternative to implantation. The objective of this study is to compare the long-term viability of percutaneously injected crushed auricular cartilage to surgically implanted cartilage in the rabbit.

**Methods:**

Auricular cartilage was harvested bilaterally in 10 New Zealand white rabbits. A 1 cm^2^ cartilage graft was implanted surgically on the upper nasal dorsum. The remaining cartilage was crushed and percutaneously injected on the lower nasal dorsum. Volume and mass of each graft were compared between pre-implantation and after 3 months of observation. A histological study was conducted to evaluate chondrocyte viability and degree of fibrosis on pre and post-implantation cartilage.

**Results:**

Mass and volume remained similar for surgically implanted cartilage grafts. Mass and volume diminished by an average of 47% and 40% respectively after 3 months for the injected crushed cartilage grafts. Chondrocyte viability was an average of 25% lower in the injected grafts.

**Conclusions:**

Cartilage injection is a promising technique that must be refined to increase chondrocyte viability. Developing an appropriate injection apparatus would improve this technique.

## Background

Nasal dorsum irregularities can result from multiple causes: trauma, surgical complications from previous rhinoplasty, cancer resection and congenital malformations [1]. Surgical repair of these defaults often requires the use of cartilage grafting. There exist many donor sites for the graft, such as the nasal septum, the conchal cartilage or costal cartilage [2]. Once harvested, the cartilage graft may be implanted whole or undergo different degrees of crushing to render it more malleable [3]. Positioning of the graft onto the nasal dorsum can be accomplished by open rhinoplasty or closed rhinoplasty.

Crushing of the cartilage prior to grafting has been found to diminish the proportion of viable chondrocytes. Ale de Souza et al. compared different degrees of cartilage crushing and found that non-crushed grafts maintained a higher area of recovered graft than crushed specimens after 120 days of implantation [4]. Noncrushed specimens also demonstrated superior chondrocyte viability than crushed grafts with statistical significance [4]. Cakmak et al. had obtained similar results in their own study, finding decreasing proportions of chondrocyte viability with increased degree of cartilage crushing [5]. Despite these findings, the increased malleability of crushed cartilage is advantageous when molding nasal dorsum defects or when the graft is delivered through injection.

Cartilage injection onto the nasal dorsum has first been studied by Limberg in 1957 [6]. He reported the existing technique which used large 1.55 mm diameter cannulas without an incision or dissection of a subcutaneous tunnel [6]. He introduced a “revolver-syringe” which allowed high pressure injection [6]. However, no other study was done until Noordzij et al. evaluated preparation techniques to produce a cartilage of an easily injectable consistency [7]. The otologic burr yielded the finest injectable slurry compared with mincing using a scalpel, the Cottle cartilage crusher or the cartilage morselizer [7]. The paste produced could easily be injected through a 16 gauge needle [7]. However, *in vivo* injection was not undertaken. No study evaluating the viability of injected cartilage grafts on the nasal dorsum has been found in the literature.

The aim of this study is to evaluate the long-term viability of injected auricular cartilage in comparison to surgically implanted cartilage on the nasal dorsum of the rabbit.

## Methods

The New Zealand white rabbit is a well-validated model for the study of cartilage grafting, especially when involving the nasal dorsum [4]. This study used 10 New Zealand white male rabbits aged approximately 3 months and weighing from 3.3 to 3.7 kg (average 3.5 kg). The research protocol was reviewed by a veterinarian and approved by the University of Montreal Deontological Committee on Animal Experimentation. Institutional guidelines regarding animal experimentation were followed.

For one week prior to surgery, the animals acclimatized to the elizabethan collars they had to wear for 10 post-operative days in prevention of self-trauma. General anesthesia was performed using Ketamine (35 mg\kg) and Xylazine (5 mg/kg). Ocular protection was provided with ointment. Breathing and heart rate were monitored by a licensed animal technician.

**Figure 1:**
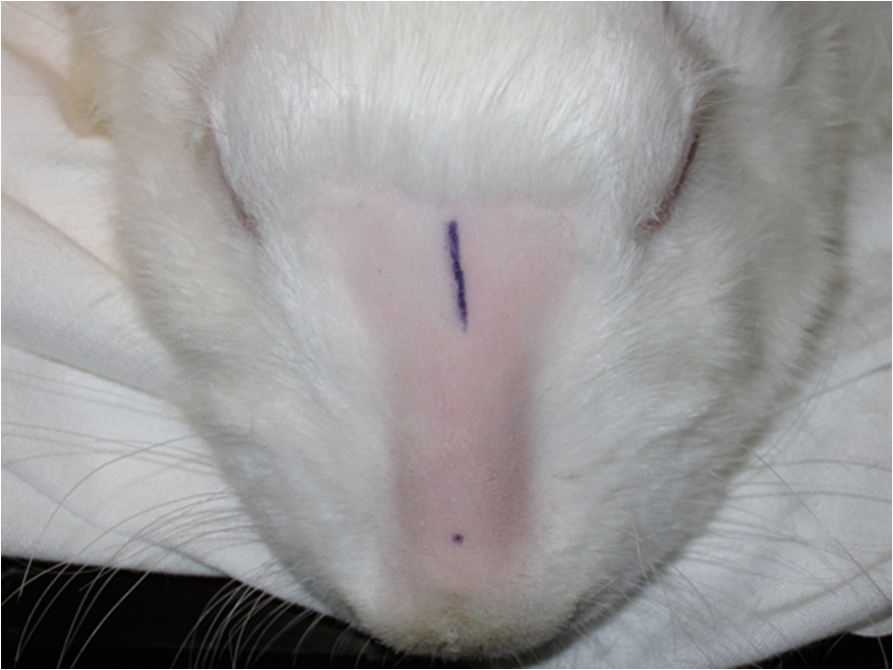
Incision for whole cartilage graft implantation and location for cartilage injection.

The nasal dorsum and ears were shaved using an electric clipper. Infiltration of the ears and nasal dorsum with xylocain 1% solution containing epinephrine 1:100,000 aided with pain control and hemostasis. Only the right ear was used for the first three rabbits, but the amount of cartilage yielded was insufficient, so both ears were used for rabbits four through ten. Surgical disinfection was done with four consecutive chlorhexidine sponges and sterile techniques were used throughout. The injection site on the lower nasal dorsum and the implantation site on the upper dorsum were marked (Figure 1).

**Figure 2:**
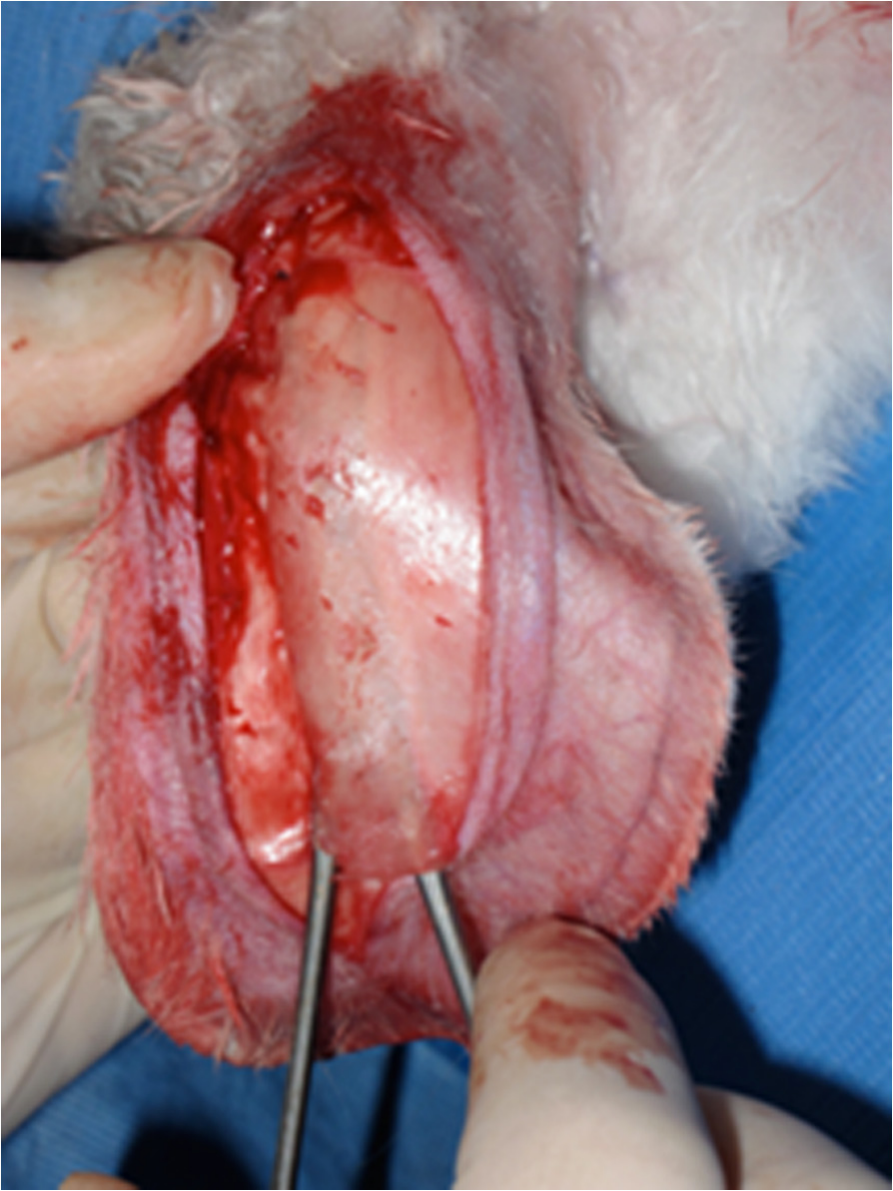
Posterior auricular incision and dissection of cartilage specimen.

Each ear was vertically incised on its posterior aspect using a scalpel, with care to avoid the main auricular vein (Figure 2). Hemostasis was done with a disposable electrical cautery. The subperichondral plane was found with a small scissor and the incision was extended in that plane along its entire length. Blunt dissection of the perichondrium to expose the cartilage was done with a hemostatic clamp (Figure 2). A rectangular cartilage window was cut with the scalpel. Care was taken to leave sufficient cartilage architecture to maintain ear support.

**Figure 3:**
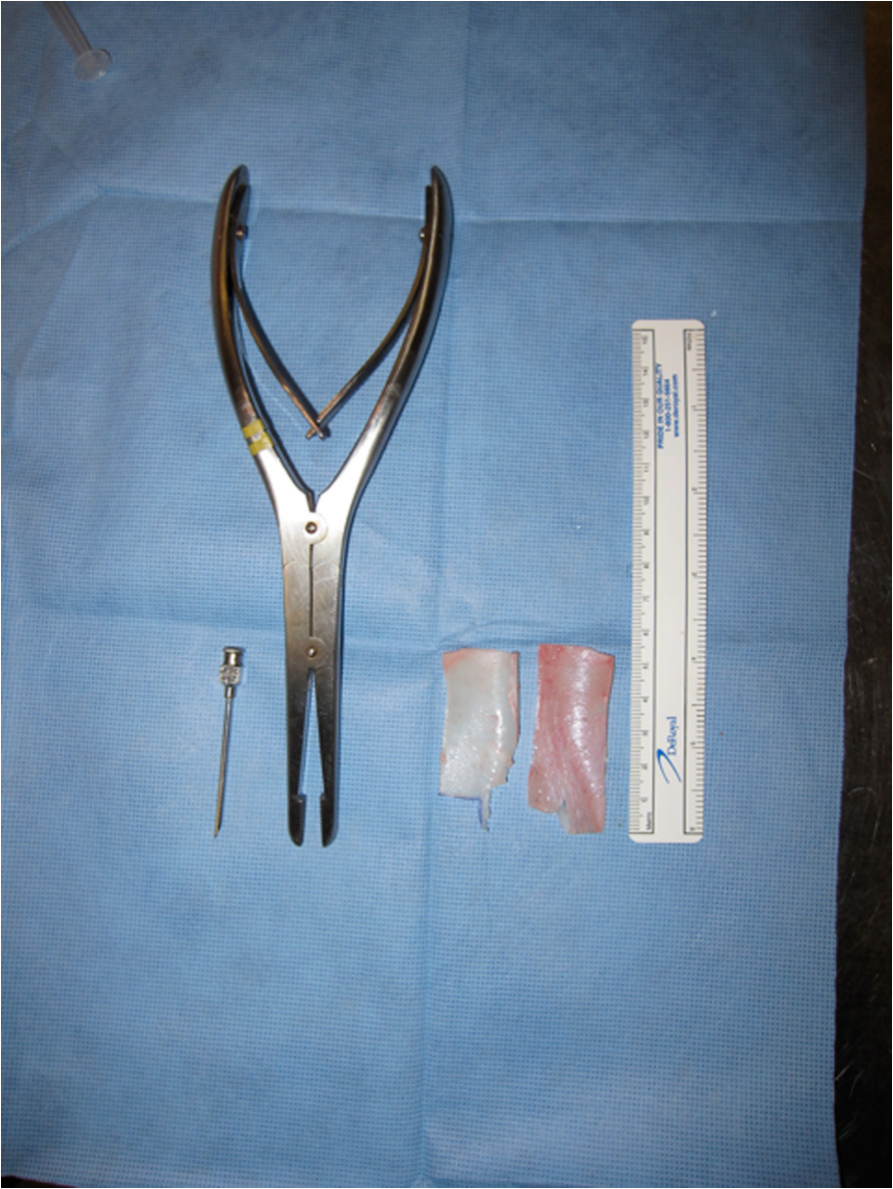
Injection cannula, cartilage morselizer, cartilage specimens from both ears and ruler (from left to right).

The anterior aspect of the cartilage rectangle was then carefully dissected to free it from the overlying perichondrium without puncturing the anterior skin flap. Once the cartilage rectangle was obtained (Figure 3), the wound was closed with a plain 4–0 continuous running suture.

**Figure 4:**
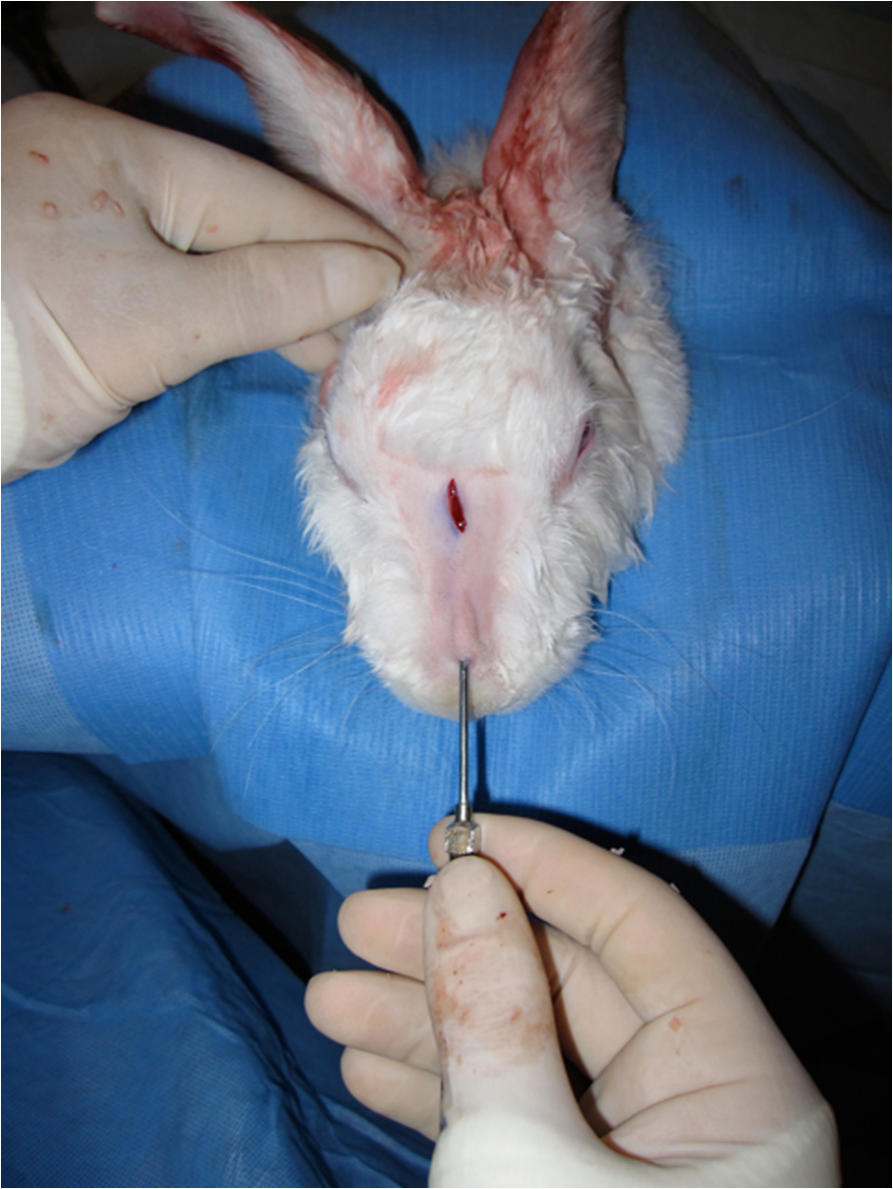
Injection of crushed cartilage graft in the inferior portion of the nasal dorsum.

A 1 cm x 1 cm whole cartilage graft was harvested from the rectangle for implantation. The graft was weighed and its volume was determined by water displacement in a 3 ml syringe. A 1.5 cm incision was made on the superior portion of each rabbit’s nasal dorsum followed by minimal dissection to create a subcutaneous pocket. The graft was then implanted and the wound was closed with plain 4–0 interrupted sutures (Figure 4).

The critical step was crushing the remaining cartilage to produce an injectable slurry. Preliminary trials were conducted prior to surgery to determine the best cartilage crushing technique. The otologic burr did not yield a satisfactory volume of cartilage and was troublesome to use. The Cottle cartilage crusher is a hinged anvil. The cartilage sample is placed between the closed moving pieces and struck with a mallet. It did not break the cartilage fibers sufficiently to render the graft injectable.

The optimal method was dicing the cartilage with a scalpel before crushing it with the cartilage morselizer. The morselizer uses serrated jaws that tear through cartilage fibers. This process yielded a cartilage paste that could pass through the injection cannula (Figure 3).

The slurry was passed successively through 1 ml syringes. Once satisfactory consistency was obtained, the apparatus was weighted with the cartilage and cannula on an analytical scale accurate to seven-decimal points. The volume of cartilage was also noted in the syringe. The skin on the inferior part of the nasal dorsum was pinched upwards before the cartilage was injected through the cannula (Figure 4). Sufficient pressure had to be applied to the syringe piston to inject the cartilage. Insufficiently crushed cartilage obstructed the cannula. This resulted in loss of cartilage volume and necessitated further crushing.

Two injection cannulas were used: a large bore cannula (2.5 mm) for the first three rabbits and a small bore (2 mm) cannula for rabbits four through ten. Injecting through the small bore cannula required more aggressive cartilage crushing than through the larger bore cannula

Injection resulted in a readily visible subcutaneous cartilage mound. A single interrupted plain 4–0 suture was placed to prevent graft extrusion. A sample of crushed cartilage was taken for the pre-implantation histological analysis. It should be noted again that only the right ear was used in the three first rabbits. In the remaining rabbits, we harvested cartilage from both ears. This provided sufficient volumes for effective injection.

Post-operative care consisted of antibiotic ointment applied onto the nasal dorsum and the ears. Supportive compressive dressings were done on the ears. Analgesia was provided by a dose of subcutaneous non-steroidal anti-inflammatories (NSAIDs) and was repeated if needed. The elizabethan collars were kept for ten days postoperatively to prevent traumatic graft extrusion or wound dehiscence. Sutures were removed prior to removal of the collars. Some post-operative complications occurred, such as spontaneous partial graft extrusion of the injected cartilage in one rabbit, auricular hematoma in two rabbits and cellulitis in one rabbit. The partially extruded graft was removed without further complications. The hematomas were drained daily until resolution and the cellulitis was treated with topical antibiotic cream.

Britt and Park compared histological analyses of implanted cartilage grafts after four weeks, eight weeks, six months and twelve months [8]. They found superior tensile strength at four and eight weeks but stabilization of the chondrocyte viability between six and twelve months [8]. Another study compared crushed cartilage viability after two, five and ten months and did not find significant variation despite degree of crushing [5]. Based on these studies, we chose a three month implantation period, which is equivalent to twelve human months [9].

During this period, no complications were encountered and all the animals thrived. The animals were then put under general anesthesia with Ketamine (35 mg/kg) and Xylazine (5 mg/kg) before they were euthanized with pentobarbital. Shaving of the nasal dorsum was followed by a vertical incision on the nasal dorsum. Both grafts and their capsules, if present, were retrieved. Post-implantation volume and mass were measured. The samples were then stored in formaldehyde to undergo post-implantation histological analysis.

The histological analysis was performed by an experienced pathologist. The stains used were hematoxylin-phloxin for chondrocyte viability and chondroid tissue appreciation and alcian blue for glycosaminoglycan content.

## Results

The non-crushed cartilage grafts were almost macroscopically identical to their pre-implantation state. The surface area was conserved and they appeared viable. A fibrous capsule was formed around these grafts. Neo-vascularisation could be seen on some of the grafts. The injected cartilage grafts were also surrounded by a fibrous capsule. The cartilaginous contents of this capsule was similar to the pre-implantation state in all but one sample, which demonstrated necrosis of the cartilage (rabbit 5).

Mass was increased by an average of 41% (median 67%) and volume was increased by an average of 22% (median 0%) during implantation for whole cartilage grafts (Table 1). Mass was decreased by an average of 47% (median 24%) and volume was decreased by an average of 40% (median 38%) for injected cartilage grafts (Table 2).

**Table 1:** Mass and volume measurements for whole cartilage grafts

**Pre-implantation**				**Post-implantation**				**Difference**			
**Mass (mg)**		**Volume (ml)**		**Mass (mg)**		**Volume (ml)**		**Mass (%mg)**		**Volume (%ml)**	
Average	Median	Average	Median	Average	Median	Average	Median	Average	Median	Average	Median
77.4	66	0.09	0.1	109	110	0.11	0.1	+41	+67	+22	0

**Table 2:** Mass and volume measurements for injected crushed cartilage

**Pre-implantation**				**Post-implantation**				**Difference**			
**Mass (mg)**		**Volume (ml)**		**Mass (mg)**		**Volume (ml)**		**Mass (%mg)**		**Volume (%ml)**	
Average	Median	Average	Median	Average	Median	Average	Median	Average	Median	Average	Median
335	310	0.35	0.32	179	235	0.21	0.2	−47	−24	−40	−38

**Figure 5:**
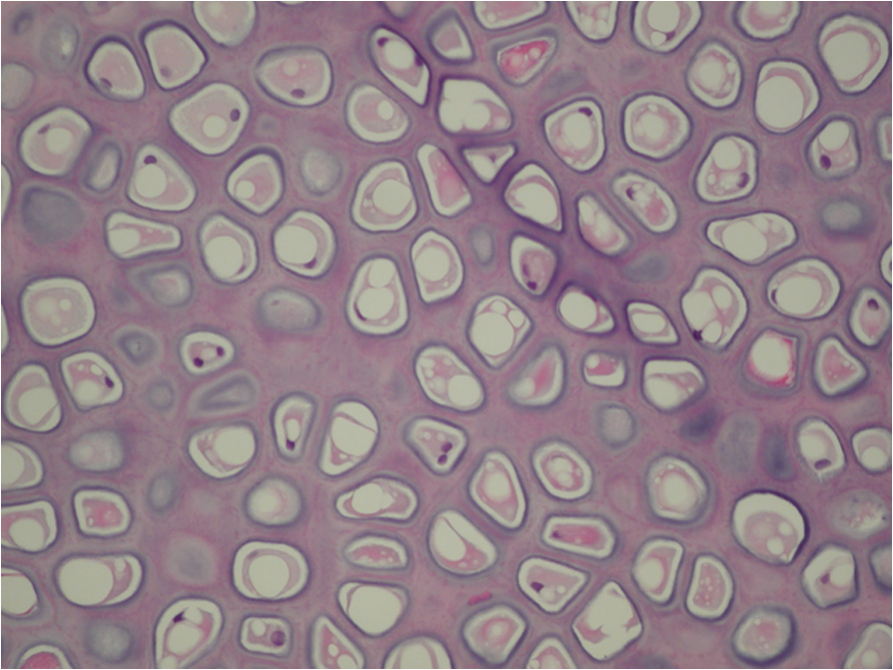
Injected cartilage graft (pre-implantation; hematoxylin-eosin, 20 X).

Histological analysis of pre-implantation whole cartilage grafts, pre-implantation injected cartilage grafts (Figure 5) and post-implantation whole cartilage grafts were all similar and showed complete chondrocyte viability and absence of fibrosis in all samples.

Histological analysis led to the exclusion of the crushed cartilage grafts from rabbits 5 and 10. Rabbit 5 demonstrated an abscess in lieu of viable cartilage and rabbit 10 showed a granulomatous foreign body reaction. The remaining grafts were separated according to the diameter of the injection cannulas.

**Figure 6:**
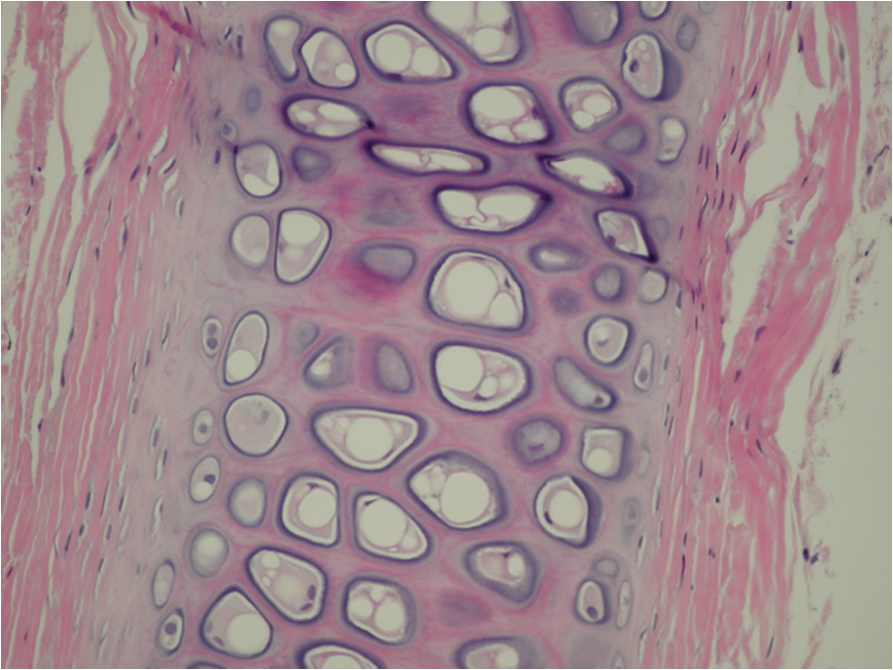
Injected cartilage graft (post-implantation, hematoxylin-eosin, 20 X).

The first three crushed cartilage grafts were injected through a 2.5 mm large bore cannula and demonstrated an average of 93% chondrocyte viability (median 90%) with an average and median limited degree of fibrosis (Table 3). The following five crushed cartilage grafts were injected through the 2 mm small bore cannula and showed an average of 64% chondrocyte viability (median 75%) with an average and median limited degree of fibrosis. The average chondrocyte viability was 75% (median 82.5%) when all injected cartilage grafts were pooled regardless of cannula diameter (Figure 6).

**Table 3:** Histological analysis for percutaneously injected cartilage

**Cannula**	**Rabbit**	**Proportion of viable chondrocytes (%)**	**Degree of fibrosis**	**Mean/median chondrocyte viability (%)**	**Mean/median chondrocyte viability (%)**
Large bore	1	100	None	93/90	75/82.5
	2	90	Moderate		
	3	90	Slight		
Small bore	4	50	Slight	64/75	
	6	90	Moderate		
	7	75	None		
	8	30	Modrate		
	9	75	None		

## Discussion

The results obtained in our study compare favorably with similarly designed studies in the literature. Ale de Souza et al. demonstrated superior surface area recovered with non-crushed cartilage grafts when compared with crushed cartilage grafts [4]. The percentage of viable chondrocytes was also greater in non-crushed cartilage grafts [4].

Cakmak et al. also found decreasing chondrocyte viability with increased degree of graft crushing [5]. The moderately, significantly and severely crushed grafts demonstrated 50%, 30% and 10% cartilage viability respectively [5]. According to the Cakmak classification system for degrees of crushed cartilage, the injected crushed cartilage in our study is severely crushed, meaning its integrity is totally destroyed [10]. The injected cartilage in our study remained 64% viable on average, with a median of 75%.

The crushed grafts injected through the 2.5 mm large bore cannula also demonstrated greater chondrocyte viability than the crushed grafts injected through the 2 mm small bore cannula. The latter necessitated more vigourous crushing to allow delivery through the smaller diameter.

Pre and post implantation histological studies used each non-crushed cartilage graft as the control for the injected crushed graft. This provides greater confidence in attributing volume, mass, change in chondrocyte viability and degree of fibrosis to the grafting procedure itself. However, the cartilage crushing technique and the injection methods used were difficult to reproduce from graft to graft. This is due mostly to the severe degree of crushing necessary to produce an easily injectable slurry which did not obstruct the cannula. Loss of mass and volume were mostly attributable to repeated filling and emptying of the syringe following additional crushing maneuvers.

In comparison to the other studies mentioned, the injected crushed cartilage grafts offer similar or superior chondrocyte viability to surgically implanted crushed grafts [4,6]. This indicates that the injection of cartilage is not detrimental to survival of chondrocytes in the same way that crushing is. Degree of graft crushing therefore seems to be the critical attribute predicting long-term chondrocyte viability. Long-term viability of crushed cartilage grafts seems to be independent from the delivery method.

## Conclusions

Cartilage injection is a promising technique that delivers crushed cartilage without subcutaneous dissection. Chondrocyte viability and degree of fibrosis in the graft are related to the degree of cartilage crushing. Delivery of the cartilage graft through injection does not seem to influence chondrocyte viability. The major caveat with this method is the poor reliability of the injection apparatus used. Development of a cartilage injection system would increase efficiency of delivery and increase reproducibility of the results.

## Competing interests

This research was funded by a 5000$ grant from Stryker as well as a 2500$ donation from the Maisonneuve-Rosemont hospital Otolaryngology foundation. These organisations have nothing to gain or lose from the publication of this article.

## Authors’ contributions

OXB has participated in the design, performed surgical manipulations, analysed the data and drafted the manuscript. AM performed the histological analysis. AR designed the study, performed surgical manipulations and helped draft the manuscript. All authors read and approved the final manuscript.
